# Methyl 5-(4-hy­droxy-3-meth­oxy­phen­yl)-2-(4-meth­oxy­benzyl­idene)-7-methyl-3-oxo-2,3-dihydro-5*H*-thia­zolo[3,2-*a*]pyrimidine-6-carboxyl­ate

**DOI:** 10.1107/S1600536811048987

**Published:** 2011-11-25

**Authors:** H. Nagarajaiah, Noor Shahina Begum

**Affiliations:** a Department of Studies in Chemistry, Bangalore University, Bangalore 560 001, India

## Abstract

In the title compound, C_24_H_22_N_2_O_6_S, a pyrimidine ring substituted with 4-hy­droxy-3-meth­oxy­phenyl is fused with a thia­zole ring. The 4-hy­droxy-3-meth­oxy­phenyl group is positioned axially to the pyrimidine ring, making a dihedral angle 85.36 (7)°. The pyrimidine ring adopts a twist boat conformation. In the crystal, O—H⋯N inter­actions result in a chain running along the *b* axis. The carbonyl O atom bonded to the thia­zole ring is involved in two C—H⋯O hydrogen-bond inter­actions forming centrosymmetric dimers; the ten- and six-membered rings resulting from these inter­actions have *R*_2_^2^(10) and *R*_1_^2^(6) motifs, respectively.

## Related literature

For pharmacological properties of pyrimidine derivatives, see: Alam *et al.* (2010 ▶). For related structures, see: Jotani *et al.* (2010 ▶). For graph-set motifs, see: Bernstein *et al.* (1995 ▶).
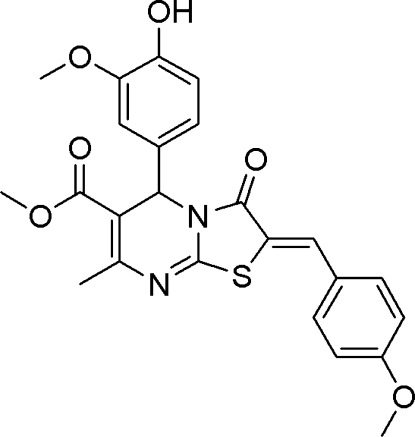


## Experimental

### 

#### Crystal data

C_24_H_22_N_2_O_6_S
*M* *_r_* = 466.50
Triclinic, 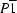

*a* = 6.8096 (12) Å
*b* = 9.9343 (18) Å
*c* = 16.246 (3) Å
α = 86.816 (3)°
β = 85.588 (3)°
γ = 81.318 (3)°
*V* = 1082.1 (3) Å^3^
*Z* = 2
Mo *K*α radiation
μ = 0.20 mm^−1^
*T* = 296 K
0.18 × 0.16 × 0.16 mm

#### Data collection

Bruker SMART APEX CCD detector diffractometer
Absorption correction: multi-scan (*SADABS*; Bruker, 1998 ▶) *T*_min_ = 0.966, *T*_max_ = 0.969
6570 measured reflections
4581 independent reflections
3452 reflections with *I* > 2σ(*I*)
*R*_int_ = 0.018

#### Refinement

*R*[*F* ^2^ > 2σ(*F* ^2^)] = 0.069
*wR*(*F* ^2^) = 0.279
*S* = 1.33
4581 reflections
303 parameters
H-atom parameters constrained
Δρ_max_ = 0.69 e Å^−3^
Δρ_min_ = −0.62 e Å^−3^

### 

Data collection: *SMART* (Bruker, 1998 ▶); cell refinement: *SAINT-Plus* (Bruker, 1998 ▶); data reduction: *SAINT-Plus*; program(s) used to solve structure: *SHELXS97* (Sheldrick, 2008 ▶); program(s) used to refine structure: *SHELXL97* (Sheldrick, 2008 ▶); molecular graphics: *ORTEP-3* (Farrugia, 1997 ▶) and *CAMERON* (Watkin *et al.*, 1996 ▶); software used to prepare material for publication: *WinGX* (Farrugia, 1999 ▶).

## Supplementary Material

Crystal structure: contains datablock(s) global, I. DOI: 10.1107/S1600536811048987/pv2473sup1.cif

Structure factors: contains datablock(s) I. DOI: 10.1107/S1600536811048987/pv2473Isup2.hkl

Supplementary material file. DOI: 10.1107/S1600536811048987/pv2473Isup3.cml

Additional supplementary materials:  crystallographic information; 3D view; checkCIF report

## Figures and Tables

**Table 1:** Hydrogen-bond geometry (Å, °)

*D*—H⋯*A*	*D*—H	H⋯*A*	*D*⋯*A*	*D*—H⋯*A*
O6—H6⋯N2^i^	0.82	2.01	2.783 (4)	156
C10—H10⋯O2^ii^	0.93	2.55	3.425 (4)	156
C12—H12⋯O2^ii^	0.93	2.67	3.499 (4)	149
C1—H1*A*⋯O6^iii^	0.96	2.57	3.444 (5)	152
C17—H17*C*⋯O2^iv^	0.96	2.47	3.429 (5)	179

Symmetry codes: (i) 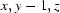; (ii) 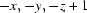; (iii) 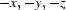; (iv) 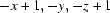.

**Fig. 1.**
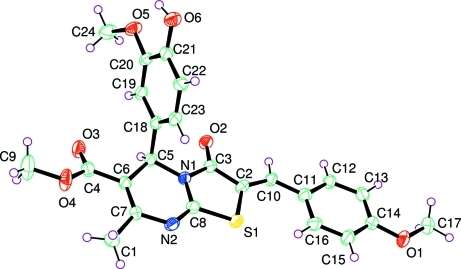
ORTEP-3 (Farrugia, 1997) view of the title compound, showing 50% probability ellipsoids and the atom numbering scheme.

**Fig. 2.**
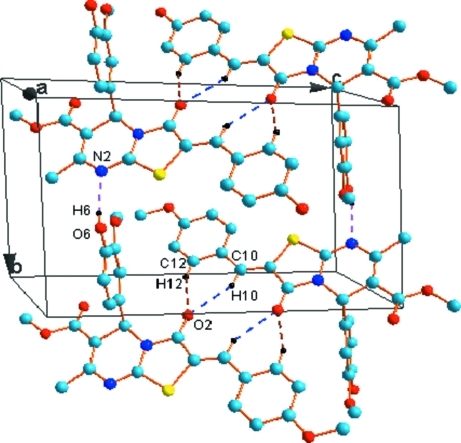
A unit cell packing of the title compound showing intermolecular interactions with dotted lines. H-atoms not involved in hydrogen bonding have been excluded for clarity.

## supplementary crystallographic information

### Comment

Pyrimidine derivatives are of interest because of their pharmacological properties (Alam *et al.*, 2010).

The central pyrimidine ring with a chiral C5 atom is significantly puckered and adopts a conformation which is best described as an intermediate between a boat and a screw boat form as seen earlier (Jotani *et al.*, 2010). The atoms C5 and N1 deviate from the mean plane C6/C7/N2/C8 by 0.1024 (3) and -0.0602 (3) Å, respectively, indicating that the conformation of the pyrimidine ring is that of a twisted boat. In the molecule, The fused thiazole-pyrimidine ring is coplanar with benzylidene ring with dihedral angle 5.19 (7)°. The dihedral angle between the thiazolopyrimidine ring and 4-hydroxy-3-methoxy-phenyl group is 85.36 (7)°. The crystal structure is stabilized by a strong intermolecular O—H···N hydrogen bond resulting in a one dimensional chain of molecules along the *b* axis. The structure is further consolidated by C—H···O type intermolecular interactions, involving carbonyl O2 atom, forming centrosymmetric dimers; the ten and 6 membered rings thus resulting from these interactions can be described as *R*^2^_2_(10) and *R*^2^_1_(6) motifs in graph-set notations (Bernstein *et al.*, 1995).

### Experimental

A mixture of 4-(4-hydroxy-3-methoxy-phenyl)-6-methyl-2-thioxo-1,2,3,4-tetrahydro -pyrimidine-5-carboxylic acid methyl ester (0.01 mol), chloroaceticacid (0.01 mol), 4-methoxy benzaldehyde (0.01 mol) and sodium acetate (1.5 g) in a mixture of glacial acetic acid and acetic anhydride (25 ml; 1:1) was refluxed for 8–10 h.The reaction mixture was concentrated and the solid thus obtained was filtered and recrystallized from ethyl acetate to get the title compound. (78% yield, mp 427 K). The compound was recrystallized by slow evaporation of an ethyl acetate-ethanol (3:2) solution, yielding pale yellow single crystals suitable for X-ray diffraction studies.

### Refinement

The H atoms were placed at calculated positions in the riding model approximation with  O—H = 0.820 Å and C—H = 0.93, 0.96, 0.98 Å, for aryl, methyl and methyne H-atoms, respectively, with *U*_iso_(H) = 1.5*U*_eq_(C) for methyl H atoms and 1.2*U*_eq_(C/O) for other H atoms.

### Crystal data

**Table:**

C_24_H_22_N_2_O_6_S	*V* = 1082.1 (3) Å^3^
*M**_r_* = 466.50	*Z* = 2
Triclinic, *P*1	*F*(000) = 488
Hall symbol: -P 1	*D*_x_ = 1.432 Mg m^−^^3^
*a* = 6.8096 (12) Å	Mo *K*α radiation, λ = 0.71073 Å
*b* = 9.9343 (18) Å	µ = 0.20 mm^−^^1^
*c* = 16.246 (3) Å	*T* = 296 K
α = 86.816 (3)°	Block, yellow
β = 85.588 (3)°	0.18 × 0.16 × 0.16 mm
γ = 81.318 (3)°	

### Data collection

**Table:**

Bruker SMART APEX CCD detector diffractometer	4581 independent reflections
Radiation source: fine-focus sealed tube	3452 reflections with *I* > 2σ(*I*)
graphite	*R*_int_ = 0.018
ω scans	θ_max_ = 27.0°, θ_min_ = 2.1°
Absorption correction: multi-scan (*SADABS*; Bruker, 1998)	*h* = −8→8
*T*_min_ = 0.966, *T*_max_ = 0.969	*k* = −12→10
6570 measured reflections	*l* = −20→20

### Refinement

**Table:**

Refinement on *F*^2^	Primary atom site location: structure-invariant direct methods
Least-squares matrix: full	Secondary atom site location: difference Fourier map
*R*[*F*^2^ > 2σ(*F*^2^)] = 0.069	Hydrogen site location: inferred from neighbouring sites
*wR*(*F*^2^) = 0.279	H-atom parameters constrained
*S* = 1.33	*w* = 1/[σ^2^(*F*_o_^2^) + (0.1558*P*)^2^] where *P* = (*F*_o_^2^ + 2*F*_c_^2^)/3
4581 reflections	(Δ/σ)_max_ < 0.001
303 parameters	Δρ_max_ = 0.69 e Å^−^^3^
0 restraints	Δρ_min_ = −0.62 e Å^−^^3^

### Special details

**Table:**

Geometry. All s.u.'s (except the s.u. in the dihedral angle between two l.s. planes) are estimated using the full covariance matrix. The cell s.u.'s are taken into account individually in the estimation of s.u.'s in distances, angles and torsion angles; correlations between s.u.'s in cell parameters are only used when they are defined by crystal symmetry. An approximate (isotropic) treatment of cell s.u.'s is used for estimating s.u.'s involving l.s. planes.
Refinement. Refinement of *F*^2^ against ALL reflections. The weighted *R*-factor *wR* and goodness of fit *S* are based on *F*^2^, conventional *R*-factors *R* are based on *F*, with *F* set to zero for negative *F*^2^. The threshold expression of *F*^2^ > 2σ(*F*^2^) is used only for calculating *R*-factors(gt) *etc*. and is not relevant to the choice of reflections for refinement. *R*-factors based on *F*^2^ are statistically about twice as large as those based on *F*, and *R*- factors based on ALL data will be even larger.

### Fractional atomic coordinates and isotropic or equivalent isotropic displacement parameters (Å^2^)

**Table:**

	*x*	*y*	*z*	*U*_iso_*/*U*_eq_	
S1	0.15410 (13)	0.32220 (9)	0.29148 (5)	0.0312 (3)	
O6	0.0905 (4)	−0.4233 (2)	0.13814 (16)	0.0352 (6)	
H6	0.0110	−0.4779	0.1447	0.053*	
O2	−0.1068 (4)	0.0292 (2)	0.39078 (14)	0.0314 (6)	
N1	−0.1155 (4)	0.1703 (3)	0.27435 (16)	0.0257 (6)	
O3	−0.6100 (4)	0.0792 (3)	0.16436 (16)	0.0372 (6)	
N2	−0.0791 (5)	0.3389 (3)	0.16660 (17)	0.0299 (7)	
O1	0.8821 (4)	0.3698 (3)	0.54235 (17)	0.0415 (7)	
C6	−0.3314 (5)	0.1959 (3)	0.15964 (19)	0.0263 (7)	
O5	−0.2673 (4)	−0.3960 (2)	0.22167 (17)	0.0365 (6)	
C5	−0.2615 (5)	0.1047 (3)	0.2339 (2)	0.0276 (7)	
H5	−0.3765	0.0977	0.2733	0.033*	
C19	−0.2694 (5)	−0.1507 (3)	0.2295 (2)	0.0276 (7)	
H19	−0.3925	−0.1391	0.2593	0.033*	
O4	−0.5284 (4)	0.1916 (3)	0.04588 (15)	0.0465 (8)	
C21	0.0002 (5)	−0.2994 (3)	0.1616 (2)	0.0288 (7)	
C18	−0.1708 (5)	−0.0377 (3)	0.20939 (19)	0.0264 (7)	
C3	−0.0444 (5)	0.1214 (3)	0.3500 (2)	0.0281 (7)	
C12	0.4787 (5)	0.1626 (4)	0.5346 (2)	0.0298 (7)	
H12	0.4232	0.0936	0.5647	0.036*	
C8	−0.0300 (5)	0.2758 (3)	0.2354 (2)	0.0281 (7)	
C20	−0.1849 (5)	−0.2791 (3)	0.20538 (19)	0.0271 (7)	
C22	0.0966 (5)	−0.1868 (3)	0.1404 (2)	0.0313 (8)	
H22	0.2194	−0.1982	0.1103	0.038*	
C2	0.1203 (5)	0.1940 (3)	0.3687 (2)	0.0297 (7)	
C10	0.2243 (5)	0.1629 (3)	0.4359 (2)	0.0276 (7)	
H10	0.1809	0.0946	0.4709	0.033*	
C11	0.3928 (5)	0.2179 (4)	0.4631 (2)	0.0315 (8)	
C7	−0.2434 (5)	0.3037 (3)	0.1307 (2)	0.0284 (7)	
C14	0.7248 (5)	0.3131 (4)	0.5199 (2)	0.0321 (8)	
C23	0.0097 (5)	−0.0576 (4)	0.1639 (2)	0.0324 (8)	
H23	0.0747	0.0168	0.1487	0.039*	
C13	0.6444 (5)	0.2068 (4)	0.5625 (2)	0.0335 (8)	
H13	0.7015	0.1654	0.6095	0.040*	
C1	−0.3048 (6)	0.4018 (4)	0.0603 (2)	0.0382 (9)	
H1A	−0.2234	0.3761	0.0112	0.057*	
H1B	−0.2881	0.4922	0.0735	0.057*	
H1C	−0.4419	0.3997	0.0514	0.057*	
C4	−0.5039 (5)	0.1509 (4)	0.1248 (2)	0.0321 (8)	
C15	0.6422 (6)	0.3702 (4)	0.4484 (2)	0.0405 (9)	
H15	0.6966	0.4408	0.4194	0.049*	
C17	0.9714 (6)	0.3169 (4)	0.6163 (2)	0.0397 (9)	
H17A	0.8779	0.3364	0.6630	0.060*	
H17B	1.0875	0.3587	0.6223	0.060*	
H17C	1.0090	0.2201	0.6135	0.060*	
C16	0.4802 (6)	0.3238 (4)	0.4194 (2)	0.0375 (9)	
H16	0.4281	0.3623	0.3708	0.045*	
C24	−0.4702 (6)	−0.3847 (4)	0.2508 (3)	0.0441 (10)	
H24A	−0.4846	−0.3583	0.3071	0.066*	
H24B	−0.5158	−0.4710	0.2476	0.066*	
H24C	−0.5479	−0.3171	0.2173	0.066*	
C9	−0.7019 (7)	0.1526 (6)	0.0106 (3)	0.0592 (14)	
H9A	−0.7048	0.0570	0.0218	0.089*	
H9B	−0.6936	0.1714	−0.0480	0.089*	
H9C	−0.8212	0.2037	0.0349	0.089*	

### Atomic displacement parameters (Å^2^)

**Table:**

	*U*^11^	*U*^22^	*U*^33^	*U*^12^	*U*^13^	*U*^23^
S1	0.0364 (5)	0.0289 (5)	0.0312 (5)	−0.0120 (4)	−0.0098 (4)	0.0032 (3)
O6	0.0355 (14)	0.0286 (13)	0.0421 (15)	−0.0088 (10)	0.0013 (11)	−0.0022 (11)
O2	0.0380 (14)	0.0306 (13)	0.0281 (12)	−0.0132 (11)	−0.0077 (10)	0.0057 (10)
N1	0.0267 (15)	0.0245 (14)	0.0270 (14)	−0.0077 (11)	−0.0056 (11)	0.0039 (11)
O3	0.0300 (14)	0.0459 (16)	0.0385 (14)	−0.0149 (12)	−0.0089 (11)	0.0070 (12)
N2	0.0384 (17)	0.0251 (15)	0.0287 (15)	−0.0107 (12)	−0.0077 (12)	0.0000 (11)
O1	0.0365 (15)	0.0447 (16)	0.0481 (16)	−0.0150 (12)	−0.0171 (12)	−0.0011 (13)
C6	0.0282 (17)	0.0274 (17)	0.0242 (16)	−0.0065 (13)	−0.0064 (13)	0.0047 (13)
O5	0.0351 (14)	0.0282 (13)	0.0480 (15)	−0.0142 (11)	0.0049 (11)	−0.0023 (11)
C5	0.0259 (17)	0.0287 (17)	0.0308 (17)	−0.0114 (13)	−0.0068 (13)	0.0036 (13)
C19	0.0250 (17)	0.0305 (18)	0.0296 (17)	−0.0111 (13)	−0.0034 (13)	0.0005 (13)
O4	0.0453 (17)	0.070 (2)	0.0309 (14)	−0.0285 (15)	−0.0194 (12)	0.0146 (13)
C21	0.0297 (18)	0.0279 (17)	0.0298 (17)	−0.0072 (13)	−0.0065 (13)	0.0030 (13)
C18	0.0270 (17)	0.0288 (17)	0.0250 (16)	−0.0072 (13)	−0.0089 (12)	0.0029 (13)
C3	0.0309 (18)	0.0264 (17)	0.0276 (17)	−0.0049 (14)	−0.0067 (13)	0.0002 (13)
C12	0.0302 (18)	0.0326 (18)	0.0280 (17)	−0.0071 (14)	−0.0057 (13)	−0.0014 (14)
C8	0.0279 (18)	0.0255 (16)	0.0308 (17)	−0.0032 (13)	−0.0015 (13)	−0.0030 (13)
C20	0.0340 (19)	0.0248 (17)	0.0253 (16)	−0.0122 (14)	−0.0055 (13)	0.0009 (13)
C22	0.0272 (18)	0.0269 (18)	0.0398 (19)	−0.0058 (13)	−0.0019 (14)	0.0028 (14)
C2	0.0319 (19)	0.0269 (17)	0.0324 (17)	−0.0098 (14)	−0.0022 (14)	−0.0064 (14)
C10	0.0296 (18)	0.0292 (18)	0.0258 (16)	−0.0086 (14)	−0.0056 (13)	−0.0011 (13)
C11	0.0317 (19)	0.0308 (18)	0.0329 (18)	−0.0074 (14)	−0.0018 (14)	−0.0001 (14)
C7	0.0281 (18)	0.0276 (17)	0.0303 (17)	−0.0035 (13)	−0.0096 (13)	0.0007 (13)
C14	0.0293 (19)	0.0285 (18)	0.0396 (19)	−0.0040 (14)	−0.0073 (14)	−0.0034 (15)
C23	0.0272 (18)	0.0298 (18)	0.043 (2)	−0.0118 (14)	−0.0066 (14)	0.0030 (15)
C13	0.0300 (19)	0.0342 (19)	0.0354 (19)	0.0006 (14)	−0.0067 (14)	−0.0023 (15)
C1	0.047 (2)	0.0300 (19)	0.039 (2)	−0.0091 (16)	−0.0150 (17)	0.0093 (16)
C4	0.0279 (18)	0.0346 (19)	0.0338 (18)	−0.0040 (14)	−0.0063 (14)	0.0029 (15)
C15	0.040 (2)	0.039 (2)	0.046 (2)	−0.0174 (17)	−0.0140 (17)	0.0082 (17)
C17	0.034 (2)	0.049 (2)	0.039 (2)	−0.0080 (17)	−0.0112 (16)	−0.0030 (17)
C16	0.044 (2)	0.0294 (19)	0.041 (2)	−0.0110 (16)	−0.0120 (17)	0.0061 (15)
C24	0.035 (2)	0.034 (2)	0.063 (3)	−0.0134 (16)	0.0078 (18)	−0.0012 (18)
C9	0.048 (3)	0.095 (4)	0.042 (2)	−0.032 (3)	−0.021 (2)	0.017 (2)

### Geometric parameters (Å, °)

**Table:**

S1—C8	1.735 (3)	C12—C13	1.387 (5)
S1—C2	1.765 (4)	C12—C11	1.388 (5)
O6—C21	1.352 (4)	C12—H12	0.9300
O6—H6	0.8200	C22—C23	1.391 (5)
O2—C3	1.210 (4)	C22—H22	0.9300
N1—C8	1.376 (4)	C2—C10	1.343 (5)
N1—C3	1.391 (4)	C10—C11	1.451 (4)
N1—C5	1.479 (4)	C10—H10	0.9300
O3—C4	1.215 (4)	C11—C16	1.416 (5)
N2—C8	1.292 (4)	C7—C1	1.503 (5)
N2—C7	1.398 (4)	C14—C15	1.386 (5)
O1—C14	1.364 (4)	C14—C13	1.389 (5)
O1—C17	1.427 (4)	C23—H23	0.9300
C6—C7	1.350 (5)	C13—H13	0.9300
C6—C4	1.479 (5)	C1—H1A	0.9600
C6—C5	1.530 (4)	C1—H1B	0.9600
O5—C20	1.369 (4)	C1—H1C	0.9600
O5—C24	1.416 (4)	C15—C16	1.382 (5)
C5—C18	1.518 (5)	C15—H15	0.9300
C5—H5	0.9800	C17—H17A	0.9600
C19—C20	1.382 (5)	C17—H17B	0.9600
C19—C18	1.404 (4)	C17—H17C	0.9600
C19—H19	0.9300	C16—H16	0.9300
O4—C4	1.338 (4)	C24—H24A	0.9600
O4—C9	1.463 (4)	C24—H24B	0.9600
C21—C20	1.392 (5)	C24—H24C	0.9600
C21—C22	1.395 (4)	C9—H9A	0.9600
C18—C23	1.378 (5)	C9—H9B	0.9600
C3—C2	1.480 (4)	C9—H9C	0.9600
			
C8—S1—C2	91.56 (16)	C12—C11—C16	117.3 (3)
C21—O6—H6	109.5	C12—C11—C10	119.2 (3)
C8—N1—C3	116.4 (3)	C16—C11—C10	123.6 (3)
C8—N1—C5	121.3 (3)	C6—C7—N2	121.8 (3)
C3—N1—C5	122.1 (3)	C6—C7—C1	127.0 (3)
C8—N2—C7	117.4 (3)	N2—C7—C1	111.2 (3)
C14—O1—C17	117.6 (3)	O1—C14—C15	115.0 (3)
C7—C6—C4	125.2 (3)	O1—C14—C13	125.7 (3)
C7—C6—C5	122.9 (3)	C15—C14—C13	119.3 (3)
C4—C6—C5	111.8 (3)	C18—C23—C22	121.2 (3)
C20—O5—C24	118.5 (3)	C18—C23—H23	119.4
N1—C5—C18	110.5 (3)	C22—C23—H23	119.4
N1—C5—C6	108.4 (2)	C12—C13—C14	119.7 (3)
C18—C5—C6	112.2 (3)	C12—C13—H13	120.2
N1—C5—H5	108.6	C14—C13—H13	120.2
C18—C5—H5	108.6	C7—C1—H1A	109.5
C6—C5—H5	108.6	C7—C1—H1B	109.5
C20—C19—C18	120.5 (3)	H1A—C1—H1B	109.5
C20—C19—H19	119.8	C7—C1—H1C	109.5
C18—C19—H19	119.8	H1A—C1—H1C	109.5
C4—O4—C9	115.8 (3)	H1B—C1—H1C	109.5
O6—C21—C20	123.0 (3)	O3—C4—O4	123.1 (3)
O6—C21—C22	118.4 (3)	O3—C4—C6	122.2 (3)
C20—C21—C22	118.6 (3)	O4—C4—C6	114.6 (3)
C23—C18—C19	118.5 (3)	C16—C15—C14	121.0 (3)
C23—C18—C5	119.9 (3)	C16—C15—H15	119.5
C19—C18—C5	121.6 (3)	C14—C15—H15	119.5
O2—C3—N1	123.1 (3)	O1—C17—H17A	109.5
O2—C3—C2	127.1 (3)	O1—C17—H17B	109.5
N1—C3—C2	109.7 (3)	H17A—C17—H17B	109.5
C13—C12—C11	122.2 (3)	O1—C17—H17C	109.5
C13—C12—H12	118.9	H17A—C17—H17C	109.5
C11—C12—H12	118.9	H17B—C17—H17C	109.5
N2—C8—N1	126.2 (3)	C15—C16—C11	120.5 (3)
N2—C8—S1	121.8 (3)	C15—C16—H16	119.7
N1—C8—S1	112.0 (2)	C11—C16—H16	119.7
O5—C20—C19	125.4 (3)	O5—C24—H24A	109.5
O5—C20—C21	113.7 (3)	O5—C24—H24B	109.5
C19—C20—C21	120.9 (3)	H24A—C24—H24B	109.5
C23—C22—C21	120.2 (3)	O5—C24—H24C	109.5
C23—C22—H22	119.9	H24A—C24—H24C	109.5
C21—C22—H22	119.9	H24B—C24—H24C	109.5
C10—C2—C3	122.8 (3)	O4—C9—H9A	109.5
C10—C2—S1	126.9 (3)	O4—C9—H9B	109.5
C3—C2—S1	110.3 (2)	H9A—C9—H9B	109.5
C2—C10—C11	131.0 (3)	O4—C9—H9C	109.5
C2—C10—H10	114.5	H9A—C9—H9C	109.5
C11—C10—H10	114.5	H9B—C9—H9C	109.5
			
C8—N1—C5—C18	108.8 (3)	N1—C3—C2—C10	−176.5 (3)
C3—N1—C5—C18	−65.4 (4)	O2—C3—C2—S1	−179.2 (3)
C8—N1—C5—C6	−14.4 (4)	N1—C3—C2—S1	3.7 (4)
C3—N1—C5—C6	171.3 (3)	C8—S1—C2—C10	177.4 (3)
C7—C6—C5—N1	10.4 (5)	C8—S1—C2—C3	−2.8 (3)
C4—C6—C5—N1	−169.6 (3)	C3—C2—C10—C11	177.5 (3)
C7—C6—C5—C18	−111.9 (4)	S1—C2—C10—C11	−2.7 (6)
C4—C6—C5—C18	68.1 (4)	C13—C12—C11—C16	−0.6 (6)
C20—C19—C18—C23	1.1 (5)	C13—C12—C11—C10	177.6 (3)
C20—C19—C18—C5	179.0 (3)	C2—C10—C11—C12	−177.3 (4)
N1—C5—C18—C23	−51.6 (4)	C2—C10—C11—C16	0.8 (6)
C6—C5—C18—C23	69.4 (4)	C4—C6—C7—N2	−179.3 (3)
N1—C5—C18—C19	130.5 (3)	C5—C6—C7—N2	0.7 (5)
C6—C5—C18—C19	−108.4 (3)	C4—C6—C7—C1	2.2 (6)
C8—N1—C3—O2	179.9 (3)	C5—C6—C7—C1	−177.8 (3)
C5—N1—C3—O2	−5.6 (5)	C8—N2—C7—C6	−9.0 (5)
C8—N1—C3—C2	−2.9 (4)	C8—N2—C7—C1	169.7 (3)
C5—N1—C3—C2	171.6 (3)	C17—O1—C14—C15	179.0 (3)
C7—N2—C8—N1	4.7 (5)	C17—O1—C14—C13	−0.7 (5)
C7—N2—C8—S1	−173.2 (2)	C19—C18—C23—C22	−2.0 (5)
C3—N1—C8—N2	−177.3 (3)	C5—C18—C23—C22	−179.9 (3)
C5—N1—C8—N2	8.1 (5)	C21—C22—C23—C18	0.9 (5)
C3—N1—C8—S1	0.8 (4)	C11—C12—C13—C14	2.3 (6)
C5—N1—C8—S1	−173.7 (2)	O1—C14—C13—C12	177.3 (3)
C2—S1—C8—N2	179.4 (3)	C15—C14—C13—C12	−2.3 (6)
C2—S1—C8—N1	1.2 (3)	C9—O4—C4—O3	4.3 (6)
C24—O5—C20—C19	13.4 (5)	C9—O4—C4—C6	−178.0 (4)
C24—O5—C20—C21	−167.5 (3)	C7—C6—C4—O3	−159.7 (4)
C18—C19—C20—O5	179.9 (3)	C5—C6—C4—O3	20.3 (5)
C18—C19—C20—C21	0.8 (5)	C7—C6—C4—O4	22.6 (5)
O6—C21—C20—O5	−0.6 (5)	C5—C6—C4—O4	−157.4 (3)
C22—C21—C20—O5	178.9 (3)	O1—C14—C15—C16	−179.0 (4)
O6—C21—C20—C19	178.6 (3)	C13—C14—C15—C16	0.6 (6)
C22—C21—C20—C19	−1.9 (5)	C14—C15—C16—C11	1.1 (6)
O6—C21—C22—C23	−179.4 (3)	C12—C11—C16—C15	−1.0 (6)
C20—C21—C22—C23	1.0 (5)	C10—C11—C16—C15	−179.2 (4)
O2—C3—C2—C10	0.6 (6)		

### Hydrogen-bond geometry (Å, °)

**Table:**

*D*—H···*A*	*D*—H	H···*A*	*D*···*A*	*D*—H···*A*
O6—H6···N2^i^	0.82	2.01	2.783 (4)	156
C10—H10···O2^ii^	0.93	2.55	3.425 (4)	156
C12—H12···O2^ii^	0.93	2.67	3.499 (4)	149
C1—H1A···O6^iii^	0.96	2.57	3.444 (5)	152
C17—H17C···O2^iv^	0.96	2.47	3.429 (5)	179

Symmetry codes: (i) *x*, *y*−1, *z*; (ii) −*x*, −*y*, −*z*+1; (iii) −*x*, −*y*, −*z*; (iv) −*x*+1, −*y*, −*z*+1.
